# Association between Parental Feeding Styles and Excess Weight, and Its Mediation by Diet, in Costa Rican Adolescents

**DOI:** 10.3390/nu14112314

**Published:** 2022-05-31

**Authors:** Kenny Mendoza-Herrera, Rafael Monge-Rojas, June O’Neill, Vanessa Smith-Castro, Josiemer Mattei

**Affiliations:** 1 Department of Nutrition, Harvard TH Chan School of Public Health, Boston, MA 02115, USA; kennymendozaherrera@g.harvard.edu (K.M.-H.); honeill@hsph.harvard.edu (J.O.); 2 Nutrition and Health Unit, Costa Rican Institute for Research and Education on Nutrition and Health (INCIENSA), Ministry of Health, Tres Rios 4-2250, Costa Rica; rmonge@inciensa.sa.cr; 3 Psychological Research Institute, Universidad de Costa Rica, City of Research, Montes de Oca 11501-2060, Costa Rica; vanessa.smith@ucr.ac.cr

**Keywords:** parental feeding styles, diet, mediation, excess weight, adolescents, Costa Rica

## Abstract

Background. Whereas parental feeding styles (PFS) influence children’s diet, less is known about this relationship in adolescents. Methods. A cross-sectional study in 686 Costa Rican adolescents (13–18 years) evaluated 4 validated PFS scores: healthy eating verbal encouragement; scolding; directly controlling diet; instrumental/emotional. Diet was evaluated through 3-day food records, deriving a Traditional Costa Rica Adolescents Diet Score (TCRAD). Excess weight (EW) measured by BMI was dichotomized following standards. Regression-based mediation analysis estimated the overall and sex-stratified odds ratios of EW for natural direct (NDE), natural indirect (NIE), and total effects (TE) of the pathway PFS→TCRAD→EW. Results. A one-unit increase in the direct control PFS score was associated with higher EW odds overall [(TE: 1.55; 95% CI: 1.04–2.31; *p-value* = 0.033), (NDE: 1.52; 95% CI: 1.02–2.27; *p-value* = 0.039)], and in boys [(TE: 2.13; 95% CI: 1.04–4.38; *p-value* = 0.039), (NDE: 2.10; 95% CI: 1.03–4.31; *p-value* = 0.042)]. Non-significant mediation by TCRAD was observed for the healthy eating verbal encouragement PFS overall (*p-value* = 0.06). Associations for the instrumental/emotional and scolding PFS were not significant. Conclusions. Direct diet control from parents may contribute to adolescents’ excess weight, particularly among boys. Parents encouraging healthy eating might support adolescents’ healthy weight through a healthy diet. Longitudinal research should clarify the association between PFS and diet-related outcomes among diverse adolescents.

## 1. Introduction

The home environment shaped by parents using specific feeding styles is critical to youth’s eating behaviors [1]. Parental feeding styles (PFS) describe parent–child interactions across food-related situations [2,3], for example, instrumental feeding (rewarding the child’s desired behavior with food), emotional feeding (snacking in response to emotional distress), parents directly controlling food intake, and encouraging children to eat healthy foods [2,4].

The influence of PFS on food intake and weight has been thoroughly studied in children <7 years old [5,6,7,8,9,10], whose marked dependence on adults causes their diet to be affected by the foods provided and their social environment [7,11]. The instrumental and emotional feeding styles are related to a lower diet quality [5,6,7,8], while encouragement to eat healthy foods is related to a healthy intake [5,6,7,8] and lower adiposity in boys [9]. The effects of parents directly controlling food intake are heterogeneous, as reflected by a positive association with overweight [10] and inverse relationships with energy-dense foods intake [5,6,7], excess weight [4,9], adiposity [12], or null results [13].

Given that adolescents have increasing personal autonomy as they grow, their interactions with parents across food-related situations may be different than at younger ages. Since autonomy is displayed as parental defiance and peer solidarity, parents may have a limited role in adolescents’ food choices [14]. However, research [1,15,16,17,18,19] proposes that parents must continue to be the primary influence on adolescent healthy eating behaviors through home rules around food [20,21,22], modeling healthy eating habits [23,24,25], availability of healthy foods at home [25,26,27], family meals [18,28,29], and an authoritative parenting style [30,31,32,33,34].

Albeit not specific to PFS, the literature suggests that parents influence adolescent diet, which mediates body weight [4]. For example, conflicts between parents and adolescents may generate emotional eating [35,36], leading to a lower diet quality [37,38]. Adolescents raised by authoritarian parents present a higher body weight [30,39,40,41], and those living with obesity are more likely to be encouraged by parents to eat healthier [42]. Similar associations are observed in Hispanic/Latino homes. For instance, homes having strict rules, marked monitoring, and high pressure to eat display obesogenic dietary choices in adolescents [43]. Parents’ coercive behaviors are associated with a higher intake of sugary drinks, sweets, and salty snacks in adolescents [44]. On the other hand, parents making vegetables more available at home and exhibiting healthier dietary choices positively influence adolescents’ diet [45].

Understanding the influence of PFS during adolescence might help stakeholders develop parenting programs to encourage healthy eating behaviors and prevent excess weight. This action is urgently needed to counteract the high prevalence of overweight and obesity among children and adolescents worldwide (just over 18%) [46]. In Latin America, this prevalence ranges from 16.6% to 35.8% in adolescents (12–19 years) [47] and represents 31% of adolescents (13–19 years) in Costa Rica [48]. While each Latin American country has a unique cultural background, they share common values, attitudes, and characteristics that influence parenting and eating. For example, the marked attitudinal familism in Latin American countries may shape food-related parent–adolescent interactions. Familism is characterized by the perception of parents serving as legitimate sources of guidance and having unquestionable authority [49], which tends to generate conflict with adolescents’ autonomy. Latin American parents generally have more and stricter rules than Euro-American families [50]. In addition, traditional Latin American cultures emphasize family rights and responsibilities based on sex [49]. Boys may be exposed to machismo-endorsing norms that grant them greater freedoms [49,51].

**Figure 1:**
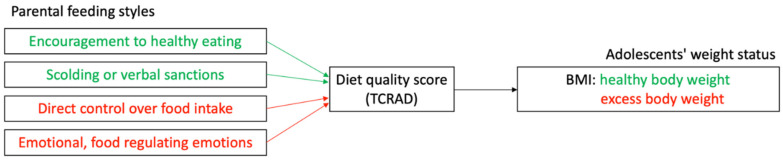
Conceptual model depicting the proposed associations between parental feeding styles (PFS) scores and adolescents’ weight status measured as body mass index (BMI) indirectly through the Traditional Costa Rica Adolescents Diet Score (TCRAD). Colors in the figure were assigned to reflect the hypothesized directionality of the proposed associations: green reflects an inverse association between PFS scores and BMI, whereas red reflects a positive association between PFS scores and BMI.

Some studies on adolescents have analyzed a single feeding style in isolation [52,53] without producing generalizable results. Although the studies in children under seven years of age suggest that the effect of PFS on body weight is mediated by dietary intakes [5,6,7,8,9,10], this phenomenon has not been quantified in adolescents. Therefore, this study aims to describe the association between PFS and excess weight in Costa Rican adolescents and ascertain if such association is mediated by diet quality. As shown in the conceptual model (Figure 1), this study formulated two hypotheses: (1) the instrumental/emotional and control feeding styles are associated with higher odds of adolescent excess weight mediated by diet quality, and (2) the encouragement to healthy eating and scolding/verbal sanctions PFS are associated with lower odds of adolescent overweight mediated by diet quality.

## 2. Materials and Methods

### 2.1. Study Population and Setting

Data pertain to a cross-sectional study carried out in adolescents aged 13–18 years and recruited in 2017 from rural and urban schools in the province of San José, Costa Rica. Details about sampling procedures, recruitment stages, and characteristics regarding the total sample have been previously described [54]. Briefly, schools were selected using a proportional-size probability method to represent urban and rural municipalities within the province [55]. A total of 10 classrooms from each school (2 from each grade from 7th to 11th) were selected by simple random sampling.

All the students in the selected classrooms were formally invited to participate in the study. Before recruitment, they were evaluated for the following inclusion criteria: (1) age of 13–18 years; (2) having mother and father living in the same household; and (3) being born in Costa Rica. Adolescents with a previous diagnosis of diabetes or other cardiometabolic diseases (self-reported/confirmed by parents) and pregnant girls were excluded from participating.

Afterward, all adolescents were provided with informed assent forms for themselves and informed consent forms for their parents. Study participants were randomly selected from those who provided signed informed consent and assent forms. Close to 5% of the initial sample chose not to participate in the study before the start, yielding a potential sample of 818 adolescents.

The parents of the adolescents recruited for the study received a questionnaire to provide information about their PFS. From the potential sample, 84% of parents (46% fathers and 54% mothers) complied. Therefore, the study’s analytic sample was 686 parents and adolescents.

The study protocol was approved by the Bioethics Committee of the Costa Rican Institute for Research and Education on Nutrition and Health (INCIENSA) under number IC-2007-01.

### 2.2. Parental Feeding Styles (PFS)

Parents were asked to fill out the Parental Feeding Style Questionnaire, designed and validated for Costa Rican adolescent populations [56]. Briefly, this psychometric scale consists of 43 items that measure 4 dimensions: encouragement of healthy eating (verbal promotion of healthy eating behaviors, 15 items); scolding/verbal sanctions (indirect control of healthy food intake, 8 items); direct control over eating (control of access to and intake of food, 9 items), and instrumental/emotional feeding (use of food to regulate emotions and behaviors, 11 items). Response options follow a 5-point Likert scale ranging from 0 (never) to 4 (always). The scale has a hierarchical structure where parental feeding styles are second-order factors; each subscale acts as an indicator. Thus, the four subscales contribute to the measured general construct. The score of each of the dimensions is the average of its items. Reliabilities for each dimension were: α = 0.92 for encouragement of healthy eating, α = 0.91 for verbal sanctions/scolding, α = 0.83 for control over eating, and α = 0.82 for instrumental/emotional feeding.

### 2.3. Anthropometric Assessment

Each participant’s height and weight were measured by trained nutritionists following the methodology described by Preedy (2012) [57]. Body mass index (BMI) values were calculated as weight in kilograms divided by height in squared meters, and body weight categories were determined using the BMI z-score for age, as recommended by the WHO [58]: <−2: underweight; ≥−2 and <+1: healthy weight (eutrophy); ≥+1 and <+2: overweight, and ≥+2: obesity. Participants with underweight (1.6%) were excluded from the present analysis. To provide a practical interpretation and correct operationalization of the outcome variable in our models, the categories of obesity (9.8%) and overweight (22.9%) were combined. Therefore, the outcome for this study was “excess weight”, defined as overweight or obesity classifications vs. “healthy weight”.

### 2.4. Diet Quality in Adolescents

To assess diet quality as a potential mediator in the association between PFS and excess weight, we used the Traditional Costa Rican Adolescent Diet Score (TCRAD), which was previously validated [59]. Briefly, the TCRAD score comprises a possible range of 0 to 14 points that reflect a consumption at or above the sex-specific median of 6 healthy food groups (legumes, vegetables, fruits, vegetable oils, dairy, and tortillas) and an intake below the sex-specific median of 8 detrimental food groups (white rice, red/processed meat, solid fats, desserts, sugary drinks, snacks, fast food, and refined bread) at one point each. This dietary score has the following cut-off points: 8–14 (high score), 6–7 (moderate score), and <6 (low score). The highest TCRAD reflects a closer adherence to a more traditional Costa Rican adolescent dietary pattern.

To determine the TCRAD score, adolescent dietary intake was assessed via 3-day food records, as previously described [60]. Briefly, students were asked to complete their 3-day food records on 2 weekdays (Monday, Tuesday, Thursday, or Friday) and 1 weekend day (Saturday or Sunday). Half of the participants were randomly selected to record the foods and drinks they consumed on Thursday, Friday, and Saturday, while the others were asked to record their intake on Sunday, Monday, and Tuesday. Data were collected during nine months of the school year (February to November), reflecting seasonal variations for Costa Rica: rainy season (May to November) and dry season (December to April). The goal was to ensure that the data captured daily and seasonal variability in food consumption. At each school, six trained nutritionists provided printed forms to the participants and instructed them on how to complete accurate food records for three consecutive days by having them write down detailed descriptions of everything they ate and drank from the time they woke up in the morning to the time they went to bed at night. The nutritionists taught the participants how to estimate serving sizes using an established manual that was developed for Costa Rica [61]. This manual includes photographs and diagrams of 3–6 serving sizes and weights for various local foods and preparations. Participants were instructed to report serving sizes using household utensils or volume and mass units. The nutritionists thoroughly reviewed the completed 3-day food records, conducting one-on-one interviews with each participant during school hours. At this interview, the nutritionists inquired about commonly missed items or ingredients (e.g., added sweeteners, added fats, candies, beverages), entered details about the types of consumed food or drinks, verified or added serving sizes, and clarified any illegible items. The nutritionists used food models, fresh foods, and various utensils to verify serving sizes.

### 2.5. Sociodemographic Variables

A paper-based questionnaire was used to collect data on sex, age, area of residence, parental education level, ownership of goods, and access to services (e.g., computers, internet, router, cable television, and water heating for the whole house), number of people in the household, and number of bathrooms in the house. To determine socioeconomic status (SES), we applied the methodology proposed by the Costa Rican National Census and Statistics Institute [62]. SES was classified into three categories: low, middle, and high.

### 2.6. Data Analysis

Categorical variables were tabulated as frequency and proportion distributions and quantitative variables as measures of central tendency (mean) and dispersion (standard deviation [SD]). Chi-square tests or Fisher’s exact tests were performed to compare the prevalence of excess weight across categories of non-quantitative variables. Means of quantitative variables pertaining to both categories of the excess weight variable were compared through Student’s *t*-tests.

Multivariate regression-based mediation analysis [63] was employed to evaluate the mediation role of the diet in the association of interest. This method enables investigators to account for a potential exposure–mediator interaction and analyze the mediation effect of continuous or binary variables. The pathway of interest was PFS scores (exposures) → TCRAD scores (mediator) → excess weight (outcome). This analysis comprises the following steps. First, regressing TCRAD (*M*) on PFS scores (*A*) and potential confounders [(*C*) sex, age, area of residence, and SES]:

$$E\{M|A=a,\;C=c\}=\beta_{0}+\beta_{1}a+\beta_{2}^{\prime }c$$

The principal linear regression coefficients represented the association between PFS scores and TCRAD (path *α*). Second, regressing excess weight (*Y*) on PFS scores, TCRAD, interaction terms of these variables, and potential confounders:

$$logit\{P(Y=1|A=a,M=m,C=c)\}=\theta_{0}+\theta_{1}a+\theta_{2}m+\theta_{3}a*m+\theta_{4}^{\prime }c$$

If the interaction terms were not statistically significant, they were removed from the model, keeping only the individual terms. The principal logistic regression coefficient reflected the association between TCRAD and the odds of excess weight (path *β*). Third, estimating the natural direct (NDE), indirect (NIE), total effects (TE), and the proportion mediated (PM) on the odds ratio scale regarding the pathway of interest by relating coefficients from paths *α* and *β* through equations proposed by Valeri and VanderWeele [63]:

$$OR^{NDE}=exp\left[\left\{\theta_{1}+\theta_{3}\left(\beta_{0}+\beta_{1}a^{*}+\beta_{2}^{\prime }c+\theta_{2}\sigma^{2}\right)\right\}\left(a-a^{*}\right)+0.5\theta_{3}^{2}\sigma^{2}\left(a^{2}-a^{*2}\right)\right],$$

$$OR^{NIE}=exp\left[\left(\theta_{2}\beta_{1}+\theta_{3}\beta_{1}a\right)\left(a-a^{*}\right)\right],$$

$$OR^{TE}=\left[\left(\theta_{1}+\theta_{3}\beta_{0}+\theta_{3}\beta_{1}a^{*}+\theta_{3}\beta_{2}^{\prime }c+\theta_{2}\beta_{1}+\theta_{3}\beta_{1}a+\theta_{3}\theta_{2}\sigma^{2}\right)\left(a-a^{*}\right)+0.5\theta_{3}^{2}\sigma^{2}\left(a^{2}-a^{*}{}^{2}\right)\right],$$

$$PM=\frac{OR^{NDE}\times \left(OR^{NIE}-1\right)}{\left(OR^{NDE}\times OR^{NIE}-1\right)}$$

The NDE refers to the association between PFS scores and the odds of excess weight, adjusted for TCRAD. The NIE showed the association between PFS scores and the odds of excess weight mediated by TCRAD. The TE refers to the composite effect of the pathway of interest and PM to an attributable percentage of the mediator to the whole association.

We replicated the regression-based mediation analysis and stratified by sex because differences in diet quality between Costa Rican boys and girls have been reported [59]. Sex was not considered a confounder in this segment of the analysis. Statistical interactions between sex and PFS, as well as between sex and TCRAD, were tested by including cross-product terms in the analog linear equations pertaining to paths *α* and *β*. As an exploration, and based on literature suggesting differences in diet quality by these factors, we also performed stratified analyses by urban vs. rural area of residence and by parent-child dyads (mother–boy, father–boy, mother–girl) [59,64,65,66]. Statistical significance was set at an *α* of 0.05, and all the analyses were carried out using SAS^®^ Studio. SAS macros developed by Valeri and VanderWeele were used to perform regression-based mediation analyses [63].

## 3. Results

A total of 33% of girls and boys had excess weight, and there were no significant differences across sex, age, area of residence, and SES (Table 1). In the bivariate analysis, encouragement of healthy eating, scolding/verbal sanctions, and instrumental/emotional PFS scores were significantly higher in parents of adolescents without excess weight than in those with this condition (Table 2). The TCRAD mean was similar between the two categories of adolescent weight, whereas the average consumption of legumes and white rice was significantly lower in adolescents with excess weight. No other significant differences regarding food group intakes between BMI categories were found. The values of continuous body weight (kg) and BMI (kg/m^2^) were higher among those adolescents with excess weight than in those with a healthy weight.

The only statistically significant exposure–mediator interaction was between the scolding PFS score and TCRAD (*β* coefficient: 0.24; *p-value*: 0.009; data not shown). Regarding the whole relation between using verbal encouragement of healthy eating behaviors and excess weight, we found a non-significant tendency for lower odds of excess weight related to each additional point of this PFS score (TE, OR: 0.74; 95% CI: 0.47, 1.17; *p-value*: 0.20) (Table 3). A unit increase in TCRAD had a non-significant mediation role of 20% in this association (NID, OR: 0.94; 95% CI: 0.87, 1.00; *p-value*: 0.06). Treating the TCRAD as a confounder (NDE) resulted in similar estimates to those observed in the whole association between the encouragement of healthy eating PFS score and excess weight (OR: 0.79; 95% CI; 0.50, 1.25; *p-value*: 0.32). In contrast, each additional point of the direct control over eating PFS score was significantly associated with higher odds of excess weight [(TE, OR: 1.55; 95% CI: 1.04, 2.31; *p-value*: 0.033), (NDE, OR: 1.52; 95% CI: 1.02, 2.27; *p-value*: 0.039)]. Diet showed no apparent mediation role in this association. No significant associations were observed for the scolding/verbal sanctions or the instrumental/emotional PFS scores.

We observed a statistically significant exposure–mediator interaction between the scolding PFS score and TCRAD in girls (*β* coefficient: 0.28; *p-value*: 0.02; data not shown). No significant exposure–mediator interactions were identified in boys. Statistically significant mediation by diet was not detected in our sex-stratified mediation analysis (Table 4). The positive association between the direct control over eating PFS score and excess weight observed in the general sample was specifically observed in boys ([TE, OR: 2.13; 95% CI: 1.04, 4.38; *p-value*: 0.039), (NDE, OR: 2.10; 95% CI: 1.03, 4.31; *p-value*: 0.042)], but not in girls. A non-significant trend for lower odds of excess weight associated with each additional point of the instrumental/emotional PFS score was identified in boys [(TE, OR: 0.44; 95% CI: 0.19, 1.05; *p-value*: 0.06), (NDE, OR: 0.43; 95% CI: 0.18, 1.02; *p-value*: 0.06)]. No significant differences were noted when stratifying results by area of residence or parent-child dyads.

## 4. Discussion

This study showed that the direct control over eating PFS was associated with higher odds of excess weight in Costa Rican adolescents, without diet quality significantly mediating this association. Stratified analyses showed that the direct adverse influence of the direct control over eating PFS on body weight was notable in boys but not in girls.

PFS represents multidimensional psychometric measures that may be conceptualized in various ways, as evidenced by other instruments to assess them [4,67]. In the questionnaire applied in this study, items under the direct control over eating subscale highlight that eating is led through parent-centric rules, similar to the conceptualization of the authoritarian PFS defined by Hughes et al. [67]. Said authors conceived the authoritarian PFS as a sub-category of parenting styles that are specific to mealtimes. Therefore, it may be assumed that the dimensions of high demandingness and low responsiveness are also applied to the control over eating PFS [68]. In accordance with our hypothesis and literature on the authoritarian parenting style [30,39,40,41], our results show that the control over eating PFS is associated with higher odds of adolescent excess weight.

Furthermore, the association between the direct control over eating PFS and excess weight was demonstrated in boys only. Gender role theory supports the notion that fathers may parent their sons and daughters differently [69], giving boys more freedom to make decisions and interact socially. Latino parents are less likely to accept adolescent autonomy [49], suggesting they may exert more direct control over boys exhibiting greater autonomy [49]. Further, parents have a more direct influence on the socialization of girls [69], making them more likely to adopt the food consumption rules established by parents. However, we did not detect significant differences in parent–adolescent sex dyads, which remains an area of further investigation.

Mediation by diet quality in the direct control–excess weight association was not detected. Adolescents using strategies to transgress parental rules and protect their autonomy [70] may help explain our findings. For example, strategies for expressing covert resistance include adolescents not communicating information about the day-to-day aspects of their lives [70], such as eating foods whose intake is controlled by parents when not under their supervision. In contrast, when adolescent boys perceive that the rules are in line with their personal benefits and autonomy, they may be more committed to following a PFS [60]. Therefore, those adolescents under controlling parents’ rules may have intentionally reported intakes that are not completely reflective of their true dietary patterns as a way to protect their autonomy. Such a hypothesis is supported by evidence pointing out that Latino-origin adolescents are increasingly likely to believe that disagreeing with parents is acceptable [71].

The instrumental/emotional feeding style may have multiple origins. Conflicts between parents and adolescents may trigger emotional eating [35,36], or parents may adopt an instrumental/emotional feeding style to reward adolescents desired behavior or reduce emotional distress to compensate for a highly controlling style [72]. Despite these differential mechanisms, studies have consistently shown that emotional eating increases unhealthy weight gain risk in adolescents [37,73]. Contrary to our hypothesis, our results showed a non-significant association between the instrumental/emotional PFS and lower odds of excess weight in boys only. Such opposite directionality may be specific to the Latin American cultural context. Therefore, more studies in countries like Costa Rica are needed to understand the role this feeding style plays in dietary choices and weight status of Latin American adolescents.

Our study noted that diet quality, measured by TCRAD, may mediate the association between the encouragement of healthy eating PFS and excess weight. Although this association was not statistically significant, a larger sample size could have led to a significant result [74]. Notably, the effect sizes yielded by our analysis and their directionality (NID, OR: 0.94; PM: 20%) are consistent with previous literature [5,6,7,8,9] and highly plausible. Results did not confirm our hypothesis regarding the scolding/verbal sanctions PFS, either. Such unexpected results may be due to cultural idiosyncrasies, for example, uncommon scolding reported among Latino-origin low-income mothers [75] or adolescents evading the indirect control characteristic of the scolding PFS as a mechanism of defense of their autonomy [70].

We acknowledge the limitations of our study. First, PFS may influence dietary habits and body weight in the medium or long term, a phenomenon that was not completely captured in our cross-sectional assessment, which also limits causal interpretations. Additionally, plausible confounders such as physical activity were not measured and incorporated into our analysis, which could introduce residual confounding. Residual confounding by this and other factors may lead to weaker effect sizes, explaining the lack of statistically significant estimates for the mediation effect of diet quality in the association of the PFS and excess weight. Lastly, undereating, as reflected by a low BMI, could not be assessed in our study due to few cases; future studies should investigate if PFS is associated with underweight status, as well.

Our study also has strengths that must be emphasized. First, diet evaluation through diet records is considered the gold standard among other dietary assessment instruments [76]. We also conducted objective procedures to operationalize the exposure (PFS) through an instrument specific to the Costa Rican population [56] and standardized methods to define the presence of excess weight in adolescents. Furthermore, while the participants may not be representative of Costa Rica as a whole, the Province of San Jose is where most of the national adolescent population (30%) is concentrated [77]. Finally, the robust mediation analyses offer an innovative approach with a well-defined exposure and outcome to study the intersection between PFS, diet, and excess weight.

## 5. Conclusions

Our study contributes evidence that PFS may play a role in the weight outcomes of adolescents in Costa Rica. Using direct control by parents over adolescents’ feeding should be discouraged because it is associated with a higher likelihood of adolescent excess weight. Conversely, healthy eating behaviors encouraged by parents might benefit Costa Rican adolescents’ weight via a healthier dietary intake. These findings must be taken into consideration when designing healthy eating interventions at the household or family level.

Large, longitudinal studies in cohorts of diverse adolescents of all genders should be conducted to clarify the exact mechanism through which PFS exerts its influence on diet and weight. Expansion of this line of research to more Latin American countries will help clarify the interconnection between PFS, diet, and excess weight in their distinctive sociocultural contexts and provide evidence with augmented generalizability to better define strategies that prevent excess adiposity in younger ages.

## Figures and Tables

**Table 1:** Sociodemographic characteristics of Costa Rican adolescents stratified by excess weight categories.

	Total		Healthy Weight		Excess Weight		*p-Value* ^1^
Variables	%	*n*	%	*n*	%	*n*	
**Overall**		686	67.1	460	32.9	226	
**Sex**							
Boys	35.4	243	67.1	163	32.9	80	
Girls	64.6	443	67.0	297	33.0	146	0.99
**Age**							
13–14 years	40.2	276	65.6	181	34.4	95	
15–16 years	36.6	251	66.5	167	33.5	84	
17–18 years	36.6	159	70.4	112	29.6	47	0.57
**Area of residence**							
Urban	48.0	329	67.5	222	32.5	107	
Rural	52.0	357	66.7	238	33.3	119	0.82
**SES**							
Low	32.9	226	71.2	161	28.8	65	
Middle	41.0	281	63.7	179	36.3	102	
High	26.1	179	67.0	120	33.0	59	0.20

^1^ χ^2^ test or Fisher’s exact test (contingency tables for more than 2 categories or proportion comparison). Abbreviations: SES, socioeconomic status.

**Table 2:** Parental feeding styles, diet quality, and anthropometric indicators in Costa Rican adolescents stratified by excess weight categories (*n* = 686).

Variables	Total		Healthy Weight		Excess Weight		
	*Mean*	*SD*	*Mean*	*SD*	*Mean*	*SD*	*p-Value* ^4^
**PFS styles scores**							
Verbal encouragement of healthy eating	2.0	0.8	2.1	0.8	1.9	0.7	0.009
Scolding (indirect control) ^1^	2.4	0.9	2.5	0.9	2.3	0.9	0.010
Direct control ^2^	1.9	0.9	1.9	0.9	1.8	0.8	0.15
Instrumental/emotional ^3^	2.3	0.7	2.4	0.8	2.2	0.7	0.009
**TCRAD score**	7.2	1.9	7.2	1.9	7.1	1.8	0.87
**Healthy TCRAD components (g/day)**							
Legumes	43.6	47.2	46.7	50.3	37.2	39.7	0.007
Vegetables	56.5	67.5	56.1	68.0	57.4	66.7	0.81
Fruits	81.2	101.9	84.1	105.8	75.3	93.6	0.27
Vegetable oils	6.7	4.1	6.9	4.2	6.3	3.8	0.08
Dairy	112.0	151.6	105.5	147.4	125.2	159.1	0.11
Tortillas	6.1	14.2	6.0	12.7	6.5	16.8	0.69
**Detrimental TCRAD components (g/day)**							
White rice	142.2	104.2	151.8	109.6	122.7	89.4	<0.001
Red/processed meat	39.7	41.8	40.2	43.0	38.7	39.2	0.67
Solid Fats	10.2	23.2	9.5	22.3	11.7	24.9	0.24
Desserts	44.7	50.8	45.0	45.0	44.0	61.0	0.83
Sugary drinks	298.8	295.5	294.3	279.2	307.8	326.7	0.59
Snacks	14.5	19.0	14.3	19.0	15.0	19.0	0.63
Fast food	38.1	63.2	38.3	64.6	37.7	60.5	0.89
Refined bread	49.5	38.4	50.9	39.2	46.8	36.8	0.19
Body weight (kg)	57.1	13.2	51.0	7.9	69.6	13.0	<0.001
Height (cm)	159.5	8.4	159.2	8.4	160.0	8.3	0.96
Body mass index (kg/m^2^)	22.3	4.3	20.0	2.1	27.1	3.7	<0.001

^1^ Verbal sanctions/scolding—indirect control of healthy food intake. ^2^ Direct control of access to and intake of food. ^3^ Use of food to regulate emotions and behavior. ^4^ Student *t*-test. Abbreviations: TCRAD, Traditional Costa Rican Adolescents’ Diet; PFS, Parental feeding styles; g, grams; kg, kilograms; cm, centimeters; m, meters; SD, standard deviation.

**Table 3:** Mediation of TCRAD score in the association between PFS scores and the odds of excess weight in Costa Rican adolescents (*n* = 686).

	Verbal Encouragement of Healthy Eating				Scolding (Indirect Control)				Direct Control				Instrumental/Emotional			
Effects	OR	95% CI		*p-Value*	OR	95% CI		*p-Value*	OR	95% CI		*p-Value*	OR	95% CI		*p-Value*
NDE ^1,2^	0.79	0.50	1.25	0.32	0.81	0.55	1.18	0.27	1.52	1.02	2.27	0.039	0.68	0.42	1.13	0.14
NIE ^1,3^	0.94	0.87	1.00	0.06	1.05	0.98	1.12	0.17	1.02	0.98	1.06	0.43	1.05	0.99	1.12	0.12
TE ^1,4^	0.74	0.47	1.17	0.20	0.85	0.57	1.25	0.40	1.55	1.04	2.31	0.033	0.72	0.44	1.19	0.20
PM	0.20				−0.25				0.04				−0.12			

^1^ We obtained natural direct effect (NDE), natural indirect effect (NIE), total effect (TE), and proportion mediated (PM) through the methods proposed by Valeri and VanderWeele [63]. ^2^ PFS scores → excess weight. ^3^ PFS scores → excess weight mediated by TCRAD scores. ^4^ PFS scores → TCRAD → excess weight. Abbreviations: TCRAD, Traditional Costa Rican Adolescents’ Diet; PFS, Parental feeding style; OR, odds ratio; CI, confidence interval.

**Table 4:** Mediation of TCRAD scores in the association between PFS scores and the odds of excess weight stratified by sex in Costa Rican adolescents (*n* = 686).

	Girls (*n* = 443)															
	Verbal Encouragement of Healthy Eating				Scolding (Indirect Control)				Direct Control				Instrumental/Emotional			
Effects	OR	95% CI		*p-Value*	OR	95% CI		*p-Value*	OR	95% CI		*p-Value*	OR	95% CI		*p-Value*
NDE ^1,2^	0.80	0.46	1.40	0.44	0.74	0.48	1.14	0.17	1.40	0.86	2.29	0.18	0.87	0.47	1.62	0.66
NIE ^1,3^	0.96	0.90	1.03	0.27	1.04	0.96	1.14	0.33	1.00	0.97	1.03	0.87	1.02	0.97	1.08	0.39
TE ^1,4^	0.77	0.44	1.35	0.37	0.77	0.50	1.19	0.24	1.41	0.86	2.29	0.17	0.89	0.48	1.66	0.71
PM	0.13				−0.14				0.01				−0.18			
	**Boys (*n* = 243)**															
	**Verbal Encouragement of Healthy Eating**				**Scolding (Indirect Control)**				**Direct Control**				**Instrumental/Emotional**			
**Effects**	**OR**	**95% CI**		***p-Value***	**OR**	**95% CI**		***p-Value***	**OR**	**95% CI**		***p-Value***	**OR**	**95% CI**		***p-Value***
NDE ^1,2^	0.62	0.28	1.42	0.26	1.08	0.61	1.93	0.79	2.10	1.03	4.31	0.042	0.43	0.18	1.02	0.06
NIE ^1,3^	0.93	0.76	1.13	0.46	1.01	0.97	1.04	0.73	1.01	0.97	1.06	0.58	1.04	0.94	1.15	0.49
TE ^1,4^	0.58	0.26	1.29	0.18	1.09	0.61	1.95	0.77	2.13	1.04	4.38	0.039	0.44	0.19	1.05	0.06
PM	0.11				0.07				0.03				−0.03			

^1^ We obtained natural direct effect (NDE), natural indirect effect (NIE), total effect (TE), and proportion mediated (PM) through the methods proposed by Valeri and VanderWeele [63]. ^2^ PFS scores → excess weight. ^3^ PFS scores → excess weight mediated by TCRAD scores. ^4^ PFS scores → TCRAD → excess weight. Abbreviations: TCRAD, Traditional Costa Rican Adolescents’ Diet; OR, odds ratio; CI, confidence interval.

## Data Availability

The data presented in this study are available on request to the corresponding author.
